# Managing patient records in the eye unit

**Published:** 2010-12

**Authors:** Ingrid Mason, Jonathan Pons

**Affiliations:** Medical Advisor, PO Box 58004, 00200 City Square, Ring Road Parklands, Nairobi, Kenya.; Ophthalmologist and Programme Director, Good Shepherd Hospital Eye Care Project, PO Box 218, Siteki, Swaziland. Email: **jono@goodshepherdhosp.org**

**Figure:**
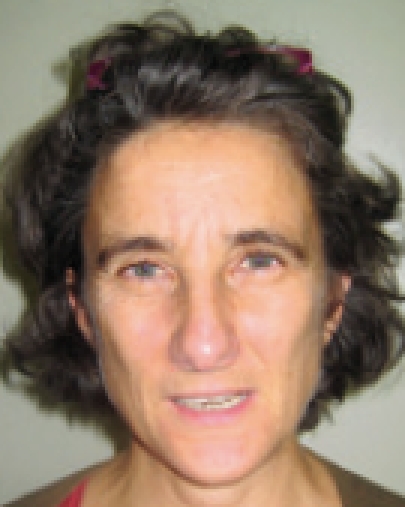


**Figure:**
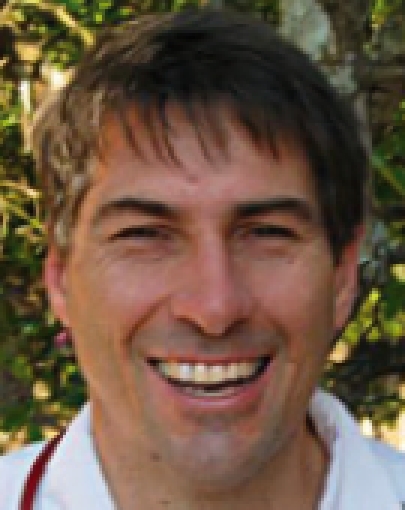


Providing excellent eye care requires excellent record keeping and data collection. Why? Because good record keeping ensures continuity of eye care, fulfils medico-legal requirements, and is professional! Good data collection, based on good record keeping, supports health management information systems (see article on page 50), hospital audit (page 48), scientific research (page 43) and provides accountability to donors.

## Types of records

**Paper records** are the standard in most low- and middle-income countries. These records can be held by the clinic or held by the patient; the latter is helpful when the health centre is unable to hold printed records or when patients are very mobile and have a choice of health care centres to attend. Sometimes there is a combination of both, as explained in the Good Shepherd Hospital case study (see box below).

**Electronic records** have many advantages because of the way data can be handled and analysed; some systems allow the eye care team to do away with paper notes altogether and enter information directly into the computer system. However, this can be expensive in terms of the equipment needed, software, and the training of staff; not to mention the cost of repairs and troubleshooting if things go wrong.

**‘Always be careful how information is stored and disposed of’**

## Organising records and keeping them safe

Information on patients is confidential. Some countries have data protection and patient confidentiality legislation which must be followed.

Patient records can be filed in different ways: by number (usually a unique patient number is assigned) by name (used less frequently), by date (date of first record), or by place (where the patient lives). Paper filing can only use one indexing system at a time; however, patient information can be indexed in multiple ways when using electronic records.

**Case study: Good Shepherd Hospital Eye Care Project**

Good Shepherd Hospital Eye Care Project in Swaziland has a hybrid system of patient- and clinic-held records, assisted by a computer-based system.

**The problem**

We inherited a paper-based system which had both patient- and clinic-held records. Frustrations with this system included the extra work required to copy information from clinic to patient record by hand. We also found that the success rate of retrieving both clinic and client records was less than 75%, which meant starting all over again! Reasons for the loss of **patient** records were many, but were generally related to the flimsy physical nature of the records. Reasons for loss of the **clinic** records were due to patient name confusion or incorrect filing, the latter due to a haphazard filing system made worse by the use of bulky stationery.

Record searching was time-consuming both for the patient and the eye care team, and loss of data made managing patients with complex eye diseases impossible.

**Finding a solution**

We asked a businessman to sit in the clinic and observe the existing records system; this ‘outsider’ perspective helped us think about and redesign the system. We also reflected on the advantages of combined patient- and clinic-held records and the need for a computer to assist with data collection.

We decided on three guiding principles in our design of a record and data keeping system:

1. **Mainly paper-based records supported by some electronic records.** Paper-based records are well understood and practical, whereas full electronic records were deemed too risky and expensive.
2. **Patient-centred and portable.** This gives patients choices about where to access eye care. By holding a card with all relevant information on it, the client also takes responsibility for their eye care.
3. **Inexpensive.** When resources are strained, the record keeping system must be economical, intuitive, meet the needs of the eye care team, and require no extra staff.

**How the system works**

The A4 cards used for clinic- and patient-held records are nearly identical and line up perfectly, so that carbon paper can be used to capture information on both records at the same time; this minimises the writing required of clinical staff. Both are A4 in size.

**Figure:**
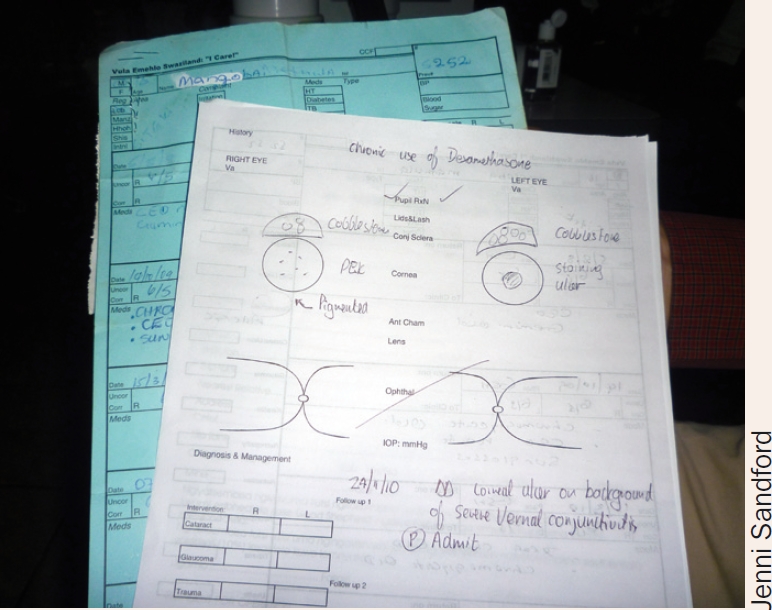
The patient-held and clinic-held records

Jenni Sandford

Sturdy card (160 grammes per square metre) is used for the hand-held patient record, which makes it less likely to get lost or damaged. Lighter card/paper (60 grammes per square metre) is used for the clinic record, which makes it easy to file.

The potential for patient name confusion is eliminated by the use of a unique patient number which is assigned to each patient as they register; this number is copied onto both the clinic and patient records.

The front page of the clinic record has space to capture demographic information at the top, space to record five consultations, a diagnosis list on the side, and space to record surgical outcomes. The patient record or card is identical, but the names and logos of the clinic's principal donors are printed in the space for surgical outcomes.

The reverse of the clinic record is printed with an eye examination schematic as well as space for listing procedures performed.

The reverse of the patient card is printed with health information as well as a hand-held Snellen chart.

At the consultation, the nurse uses carbon paper between the clinic and patient records and writes down the visual acuity, blood pressure, and blood sugar (if indicated) on both records. The doctor records the examination on the reverse of the clinic record and, using carbon paper, records diagnosis, management, and treatment on both the clinic and patient records.

The patient card is folded in half, placed in a plastic sleeve, and given to the patient, who uses it to pay fees and/or collect any prescriptions.

At the end of the day, or later that week, the nurse will enter information from the clinic-held record into the computer database. This includes the demographic information (if new, or if there are changes) and the data from the consultation (such as diagnosis and treatment). The clinical notes are not copied. This process takes about two minutes per new record, or less for a follow-up consultation.

The clinic record is then filed according to the unique client number in an ordinary lever-arch A4 file.

At follow-up, patients present their patient card and the number is used to retrieve the clinic record. In case the patient card is lost, the computer is used to look up the unique number. Staff then find the clinic record and issue a duplicate patient card.

**Figure:**
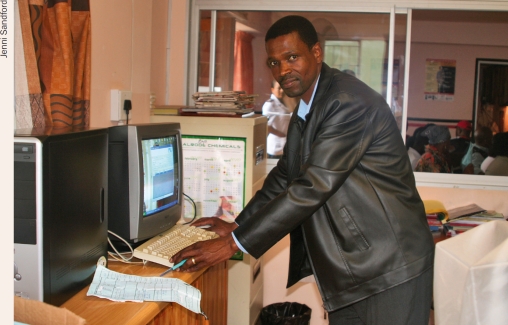
At the end of the day, or later in the week, information from the clinic-held record is entered into the computer database. SWAZILAND

Jenni Sandford

**The software**

A well-known database programme is used to capture the information on computer and the data entry fields have been designed to match the clinic-held record exactly. The programme is easy to use and does not require prior computer skills.

The same data could also be stored in a simple spreadsheet.

So far, an extra person (and therefore salary) has not been needed to record and subsequently enter data into the electronic database.

**Value and effectiveness**

The success rate of uniting patient with clinic records is now better than 95%. The system is easy to understand and operate by non-specialist eye care staff and highly accurate data can be obtained at low cost.

From the software, useful management information can be extracted, such as daily, weekly, or monthly statistics, as well as payment information for accounting purposes.

Some of the data collected, such as visual outcome after surgery and presentation by diagnosis, is used for clinical audit. Interesting research questions can also be answered, such as: “What is the average age and blood sugar of patients presenting with proliferative diabetic retinopathy?” This information is strategic in the design of public health interventions, as well as being of interest to the international medical community.

This is a work-in-progress and refinements are constantly being made, even after eight years of use.

Patient records must be kept in a safe, secure, fireproof environment that allows for expansion; as the unit becomes busier the number of patient notes that need to be kept will increase.

Always be careful how information is stored and disposed of; some countries have legislation which determines for how long patient files need to be kept.

Apart from safety concerns, there are no set rules for where records are stored. More important is to have a system that works and is efficient at retrieving patients' notes; also important is to have staff who understand the system and know how to file and retrieve notes in a systematic way.

If a member of staff takes a patient record away with them, a ‘taken by’ note should be placed in the filing system, allowing everyone else to know where the records have gone and who took them.

## Keeping track of test results

The results of any tests or investigations that a patient undergoes should be kept with the patient's other notes.

The patient's registration number (if used) must be recorded on all investigation and result slips.

Ensure that the results are kept together with other notes, whether by stapling or by using a paper punch and then linking the results and notes together.

Some hospitals use patient name plates (or sticky labels) that can be stuck to investigation slips; these reduce the risk of mixing up or losing results.

In some hospital environments, the test results are also copied into the patient notes. If this is the case, accuracy is very important!

## Other patient records

The record of care that the doctor makes regarding a patient in the ward or operating theatre should be kept together with the nursing notes. Consent forms, anaesthestic forms, treatment charts, etc. should all clearly state the patient's full name and his/her patient number.

X-rays, due to their bulk, are difficult to store with patient records. On the outside of the patient notes a coloured shape (e.g. a sticker) can alert clinical staff to the fact that X-rays are available. X-rays can then be stored in the X-ray department or in the clinical records department.

## A records department

Some hospitals may have records departments with records staff whose purpose is to collect and file records once a patient is discharged. Records staff should also be able to search for X-rays or other investigations that may not yet be filed with the patient's notes.

Record keeping courses are available in some countries through the ministry of health. Having a well-run records department saves a great deal of valuable time and gives the patient better service.

